# Analysis of microbiota in the stomach and midgut of two penaeid shrimps during probiotic feeding

**DOI:** 10.1038/s41598-021-89415-w

**Published:** 2021-05-11

**Authors:** Kentaro Imaizumi, Sasiwipa Tinwongger, Hidehiro Kondo, Ikuo Hirono

**Affiliations:** 1 Laboratory of Genome Science, Graduate School of Marine Science and Technology, Tokyo University of Marine Science and Technology, 4-5-7 Konan, Minato, Tokyo, 108-8477 Japan; 2 Department of Fisheries, Kasetklang, Chatuchak, Bangkok, 10900 Thailand

**Keywords:** Microbiology, Molecular biology, Zoology, Diseases, Pathogenesis

## Abstract

In mammals, the intestine harbors numerous bacteria that play an important role in health. Intestinal microbiota have also been thought to be an important factor in the health of shrimp. However, the barrier systems of the digestive tracts of shrimp seem to be different from those of mammals. In this study, we analyzed the bacterial composition in the stomach and midgut of two species of shrimp during administration of a probiotic, *Bacillus amyloliquefaciens* strain TOA5001 by analysis of 16S rRNA genes with Illumina sequencing technology. Whiteleg shrimp *Litopenaeus vannamei* were observed under laboratory conditions and kuruma shrimp *Marsupenaeus japonicus* were observed in an aquaculture farm. The diversities of bacteria in the stomachs of both shrimps were significantly higher than those in the midgut. Also, the microbiota changed during probiotic feeding. Feeding whiteleg shrimp the probiotic after being challenged with an acute hepatopancreatic necrosis disease (AHPND)-causing strain of *Vibrio parahaemolyticus* increased their survival compared to the control group, which suggested that the probiotic prevented AHPND. These results appear to show that a probiotic can affect the microbiota throughout digestive tract of penaeid shrimps and that probiotic can have a role in preventing disease.

## Introduction

The intestine harbors numerous bacteria that form a complicated community. Intestinal microbiota play roles in the induction of immunity, and form a biological barrier against the growth and colonization of pathogens^1^. Although these findings were mainly obtained from studies in mammals, it is expected that they will also be true for aquatic organisms^2^.

The barrier systems of the digestive tracts in mammals and other organisms are different. Invertebrates lack gastric acid in their stomach^3^. Most arthropods construct a physical barrier composed of chitin in their midgut called a peritrophic matrix (PM)^4,5^. This suggests that the intestinal microbiota that colonize the gut epithelia is a derived feature of mammal, which lacked in other organisms^6^, including crustacean^7^.

Penaeid shrimps, such as whiteleg shrimp *Litopenaeus vannamei* and kuruma shrimp *Marsupenaeus japonicus* are the most widely farmed crustacean species in the world, accounting for more than the half of the global crustacean aquaculture production^8^. Infectious diseases have emerged and spread worldwide, resulting in economic damage and threatening sustainable aquaculture and food production^9,10^. The use of antibiotics is highly restricted because of the risk of antibiotic-resistance among bacteria^11,12^. Therefore, an understanding of immunity and the barrier system of shrimp is needed to develop effective methods to protect shrimp from pathogens.

Acute hepatopancreatic necrosis disease (AHPND) [also referred to as early mortality syndrome (EMS)] has caused huge economic losses in shrimp farming. Since the first report of its occurrence in China (2009), AHPND has expanded worldwide, but especially in Asia and South America^13–16^. The main causative agents of AHPND are strains of *Vibrio parahaemolyticus* that contain a plasmid that encodes toxin genes^17–20^. The AHPND-causing strains of *V. parahaemolyticus* colonize the stomach of shrimp and then invade the hepatopancreas^21,22^. This suggests that microbiota in the stomach are related to the occurrence of AHPND^23^.

Probiotics means ‘live microorganisms, which when consumed in adequate amounts, confer a health benefit for the host’, according to a 2001 FAO report^24^. Probiotics have the potential to prevent disease in shrimp^25,26^. Some probiotic strains were shown to modulate intestinal microbiota and shrimp resistance to bacterial pathogens^27,28^. However, to the best our knowledge, there have been no studies of the effects of probiotics on the microbiota of the shrimp stomach.

Next generation sequencing of 16S ribosomal RNA (16S rRNA) gene can rapidly determine bacterial composition in a given environment^29^. Understanding a bacterial community by isolation and culture is impractical because most bacterial species are unculturable^30^. The application of next generation sequencing techniques for shrimp is thought to elucidate shrimp–bacteria interaction^31^.

Here we investigated the effects of probiotics on the bacterial composition and dynamics in the stomach and midgut of whiteleg shrimp and kuruma shrimp. Most analyses of microbiota examined only the intestine and little information is available on the stomach. The interactions between shrimp and bacteria are also unclear. Fundamental knowledge of the bacteria in the digestive tract of penaeid shrimps can be helpful not only for sustainable aquaculture production, but also to better understand the evolution of host–bacteria interactions in the digestive tract.

## Results

### Microbiota of the digestive tract of whiteleg shrimp during probiotic feeding

The copy numbers of 16S rRNA gene are not the same among different bacterial species^32^. This might affect the results of analyses of microbiota, so that the relative frequency would not indicate the actual bacterial number. However, the results are still useful for identifying the bacteria that are present and for comparing the communities before and after probiotic feeding.

**Figure 1:**
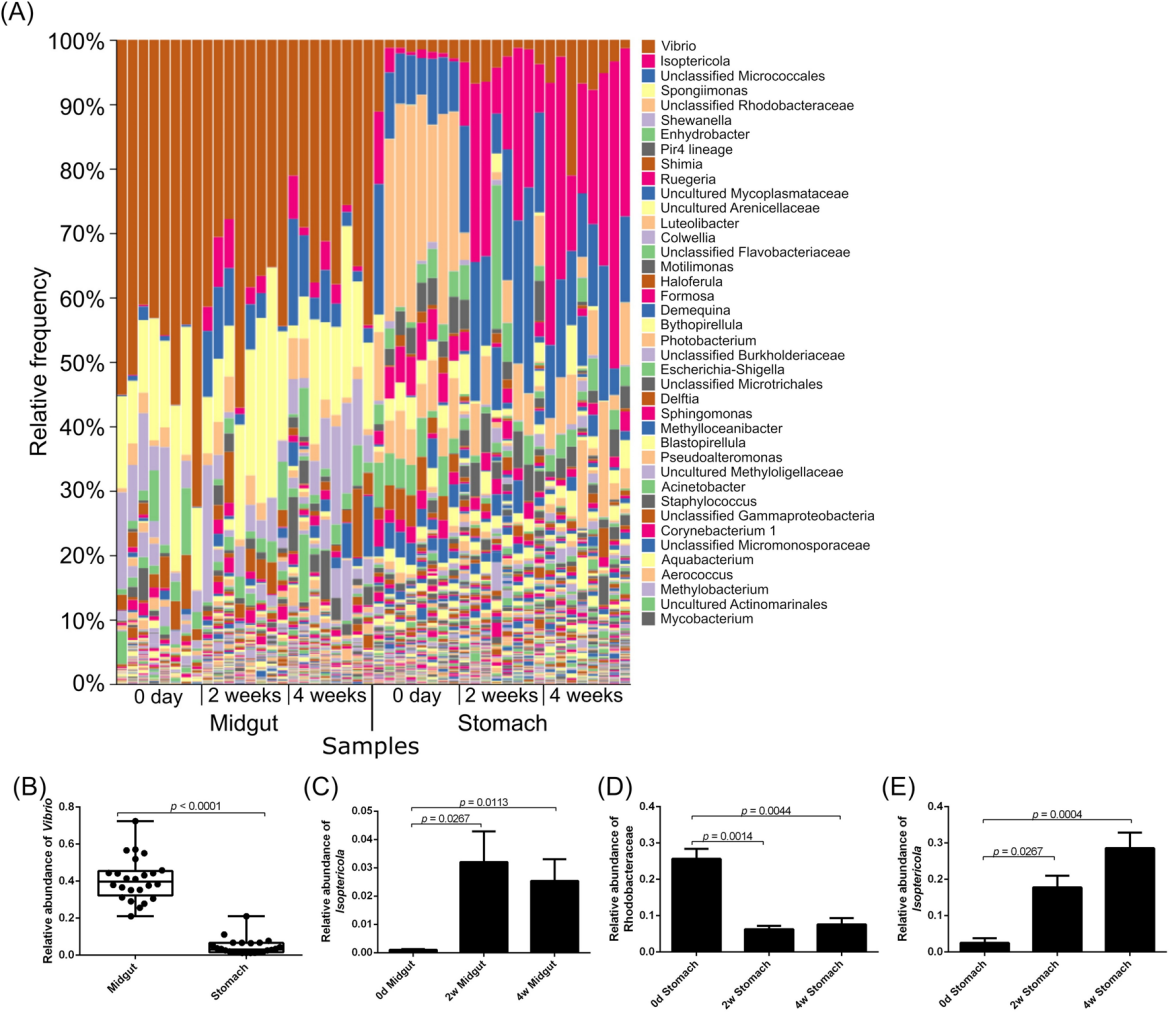
Comparison of bacterial composition of the stomach and midgut of whiteleg shrimp during probiotic-feeding in a laboratory condition. Bacterial composition (**A**). Relative abundance of *Vibrio* spp. in the midgut and stomach (**B**). Individual values are shown and the box delimits the 25th and 75th percentiles, the line in each box indicates the median, and the whiskers indicate the lowest and highest values (n = 24, Mann–Whitney test). Relative abundances of *Isoptericola* spp. in the midgut (**C**), Rhodobacteraceae bacteria in the stomach (**D**) and *Isoptericola* spp. in the stomach (**E**) of whiteleg shrimp during probiotic-feeding. Bars show the mean ± SEM (n = 8). DNA was extracted from stomach and midgut (including the shrimp tissues). Statistical analysis was performed using Dunn’s multiple comparison test following Kruskal–Wallis test.

**Figure 2:**
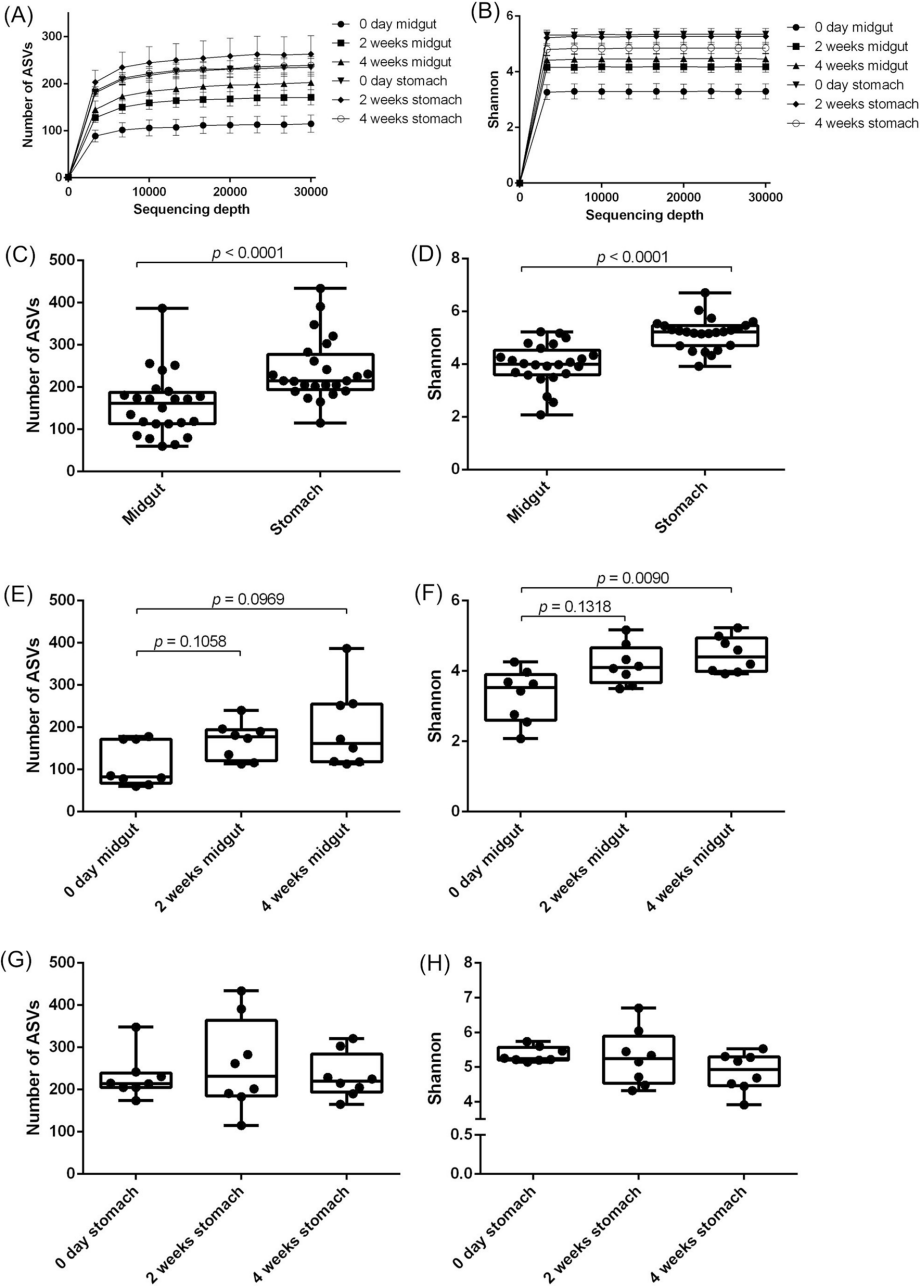
Alfa diversity of microbiota of the stomach and midgut of whiteleg shrimp during probiotic-feeding. Rarefaction curves of ASVs (**A**) and Shannon index (**B**). Bars show the mean ± SEM (n = 8). Number of ASVs (**C**) and Shannon index (**D**) were used to estimate the bacterial diversity of each organ. Individual values are shown and the box delimits the 25th and 75th percentiles, the line in each box indicates the median, and the whiskers indicate the lowest and highest values (n = 24, Mann–Whitney test). Number of ASVs (**E**) and Shannon index (**F**) of microbiota of midgut during probiotic-feeding. Number of ASVs (**G**) and Shannon index (**H**) of microbiota of stomach during probiotic-feeding. Individual values are shown and the box delimits the 25th and 75th percentiles, the line in each box indicates the median, and the whiskers indicate the lowest and highest values (n = 8). Statistical analysis was performed using Dunn’s multiple comparison test following Kruskal–Wallis test.

We analyzed bacterial composition of the midgut and stomach of whiteleg shrimp during probiotic-feeding. In whiteleg shrimp raised under laboratory conditions, the bacterial composition of midgut and stomach were different (Fig. 1A), and *Vibrio* bacteria were abundant in the midgut but not in the stomach (Fig. 1B). Two and four weeks of probiotic feeding increased the relative frequency of *Isoptericola* bacteria in the midgut (*p* = 0.0267, *p* = 0.0113) (Fig. 1C). Probiotic feeding reduced the relative frequency of Rhodobacteraceae in the stomach (*p* = 0.0014, *p* = 0.0044) (Fig. 1D). Also, probiotic feeding increased the relative frequency of *Isoptericola* bacteria in the stomach (*p* = 0.0267, *p* = 0.0004) (Fig. 1E). Rarefaction curves showed that the number of ASVs reached plateau at around 6000 of the sequencing depth (Fig. 2A) and Shannon index reached plateau at less than 3000 of the sequencing depth (Fig. 2B). Surprisingly, the number of ASVs and Shannon index of bacterial diversity in stomach were significantly higher than those in the midgut, whether or not the shrimp were fed probiotics (*p* < 0.0001) (Fig. 2C,D). No significant difference of the number of ASVs in midgut was found after two weeks and four weeks of probiotic feeding (*p* = 0.1058 and *p* = 0.0969, respectively) (Fig. 2E). The difference of Shannon index of midgut after two weeks of probiotic feeding was not statistically significant (*p* = 0.1318), however that of after four weeks was significant (*p* = 0.0090) (Fig. 2F). The diversity of microbiota in the stomach were not different among the different experimental groups (Fig. 2G,H). These results suggest that the biological diversity of microbiota in whiteleg shrimp is higher in the stomach than in the midgut, and that probiotic feeding might affect the bacterial composition throughout the digestive tract and the bacterial diversity of midgut.

### Microbiota of the digestive tract of kuruma shrimp during probiotic feeding

**Figure 3:**
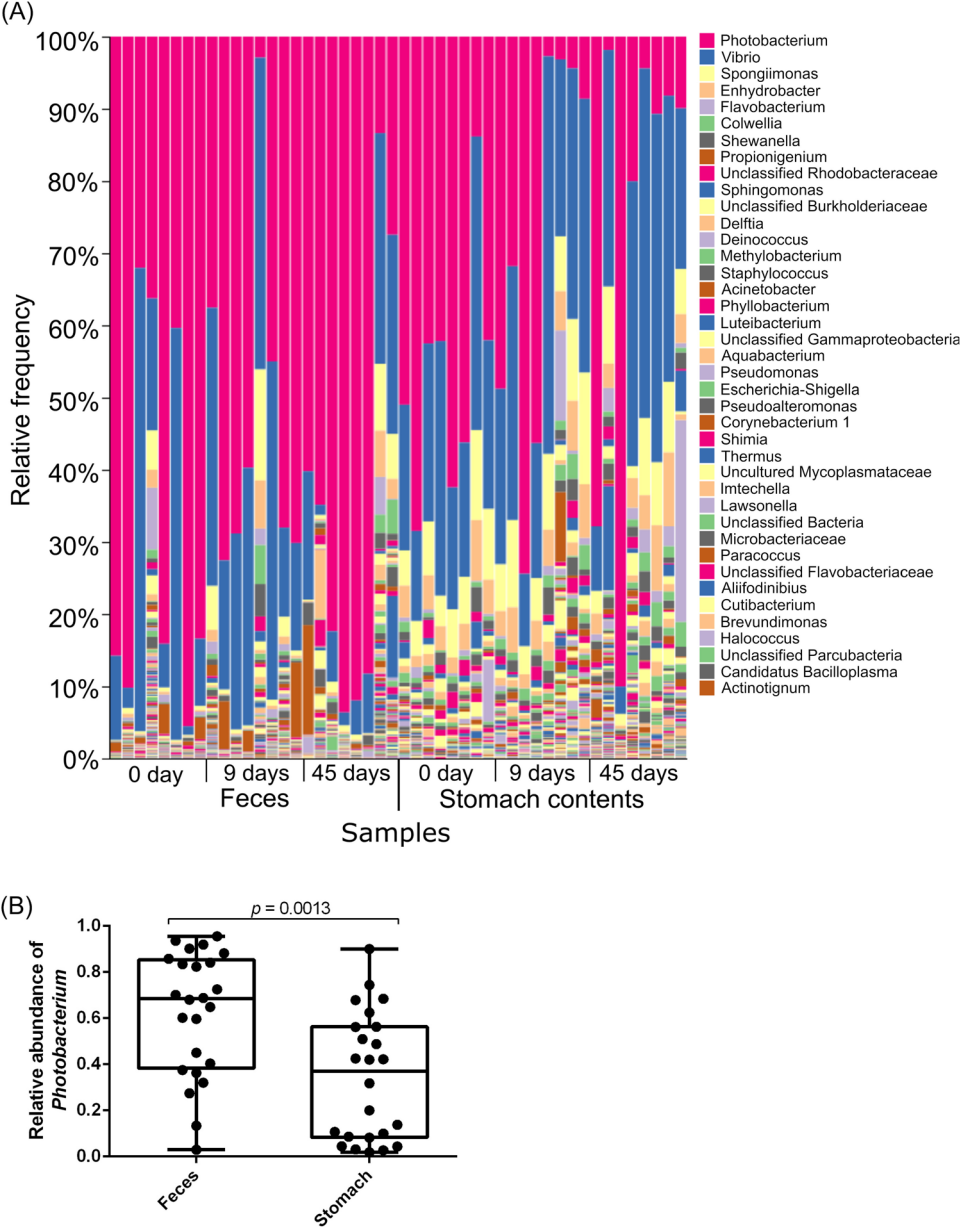
Comparison of bacterial composition of the feces and stomach contents of kuruma shrimp during probiotic-feeding in an aquaculture farm. Bacterial composition (**A**). Relative abundance of *Photobacterium* spp. in feces and stomach contents (**B**). DNA was extracted from feces and stomach contents of the shrimp (not including shrimp tissues). Shrimp was harvested at shrimp farm and transported to the laboratory, then dissected. The transportation took almost 1 day. Individual values are shown and the box delimits the 25th and 75th percentiles, the line in each box indicates the median, and the whiskers indicate the lowest and highest values (n = 24, Mann–Whitney test).

**Figure 4:**
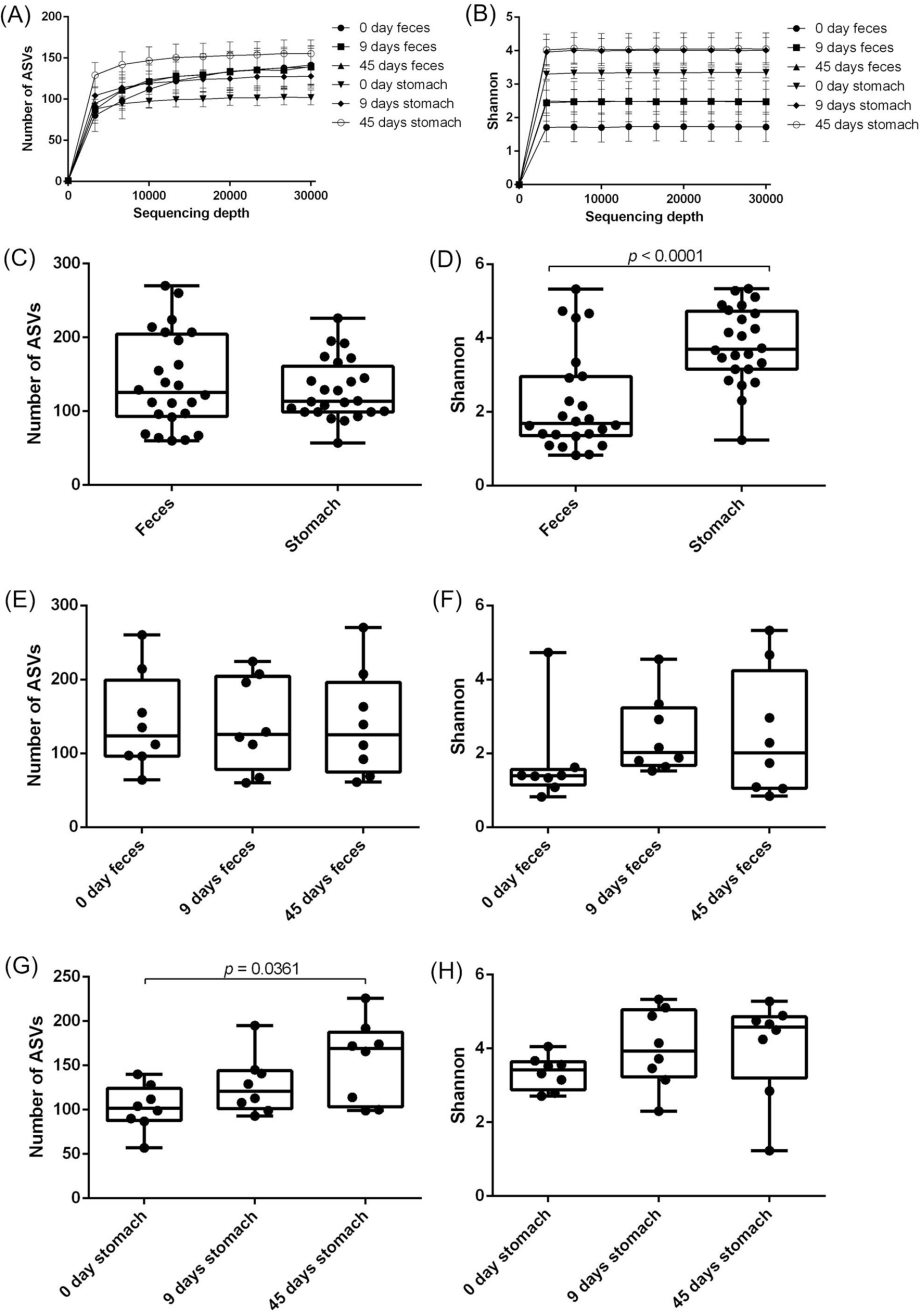
Alfa diversity of microbiota of the feces and stomach contents of kuruma shrimp during probiotic-feeding. Rarefaction curves of ASVs (**A**) and Shannon index (**B**). Bars show the mean ± SEM (n = 8). Number of ASVs (**C**) and Shannon index (**D**) were used to estimate the bacterial diversity of each organ. Individual values are shown and the box delimits the 25th and 75th percentiles, the line in each box indicates the median, and the whiskers indicate the lowest and highest values (n = 24, Mann–Whitney test). Number of ASVs (**E**) and Shannon index (**F**) of microbiota of midgut during probiotic-feeding. Number of ASVs (**G**) and Shannon index (**H**) of microbiota of stomach during probiotic-feeding. Individual values are shown and the box delimits the 25th and 75th percentiles, the line in each box indicates the median, and the whiskers indicate the lowest and highest values (n = 8). Statistical analysis was performed using Dunn’s multiple comparison test following Kruskal–Wallis test.

To analyze the microbiota of the digestive tract and the effect of probiotic on another species of penaeid shrimp, we performed an analysis of microbiota of the digestive tract of kuruma shrimp in aquaculture farm. In kuruma shrimp raised in an aquaculture pond, the bacterial composition of feces and stomach contents were different (Fig. 3A), and the relative abundance of *Photobacterium* bacteria were higher in the feces of kuruma shrimp compared to the stomach contents (*p* = 0.0013) (Fig. 3B). Rarefaction curves showed that the number of ASVs of stomach contents reached plateau at around 6000 of the sequencing depth, however, the number of ASVs of feces increased until around 20,000 of sequencing depth (Fig. 4A). Shannon index reached plateau at less than 3000 of the sequencing depth (Fig. 4B). No significant difference of the number of ASVs in the feces and stomach contents was observed (Fig. 4C). However, the Shannon index of the diversity of microbiota was significantly higher in the stomach contents than in the feces (*p* < 0.0001) (Fig. 4D). No significant difference of alfa diversity of microbiota of feces was observed (Fig. 4E,F). The number of ASVs of the stomach contents of kuruma shrimp after 45 days of probiotic-feeding were higher than the shrimp before probiotic-feeding (*p* = 0.0361) (Fig. 4G). No significant difference of Shannon index of microbiota of stomach contents was observed (Fig. 4H).

The kuruma shrimp used in the above analyses were transported from the farm to the laboratory at low temperature. We cannot rule out the possibility that the transport, which took about one day, affected the microbiota in the digestive tract.

### Principal coordinates analysis (PCoA) of the bacterial community

**Figure 5:**
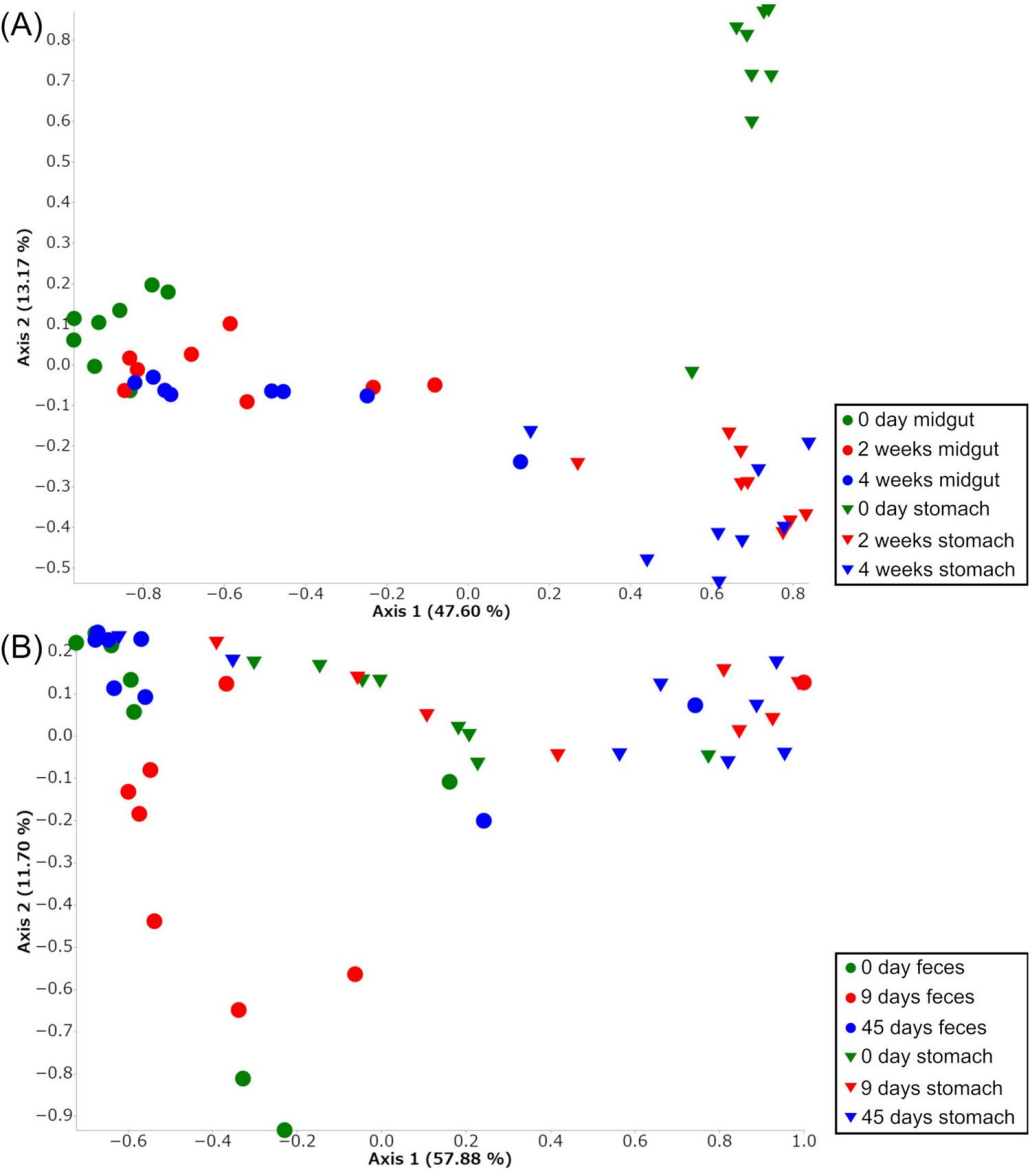
Principal coordinate analysis (PCoA) using Bray–Curtis distance of microbiota of the stomach and midgut of whiteleg shrimp (**A**) and the feces and stomach contents of kuruma shrimp during probiotic-feeding (**B**). In (**A**), spheres indicate samples of midgut and cones indicate stomach, and green indicates 0 day, red indicates 2 weeks and blue indicates 4 weeks of probiotic feeding. In (**B**), spheres indicate samples of feces and cones indicate stomach contents, and green indicates 0 day, red indicates 9 days and blue indicates 45 days of probiotic feeding. (n = 8 for each group).

The result of PCoA using Bray–Curtis distance of microbiota of whiteleg shrimp showed that the samples of midgut and stomach, and samples of each time-point of probiotic-feeding were grouped separately (Fig. 5A). The result of PCoA of microbiota of kuruma shrimp showed that the samples of feces at 9 days of probiotic-feeding were different from at day 0, and samples of stomach contents at 45 days of probiotic-feeding were different from at day 0 (Fig. 5B).

### Inhibition activity and stability of *B. amyloliquefaciens* strain TOA5001

**Figure 6:**
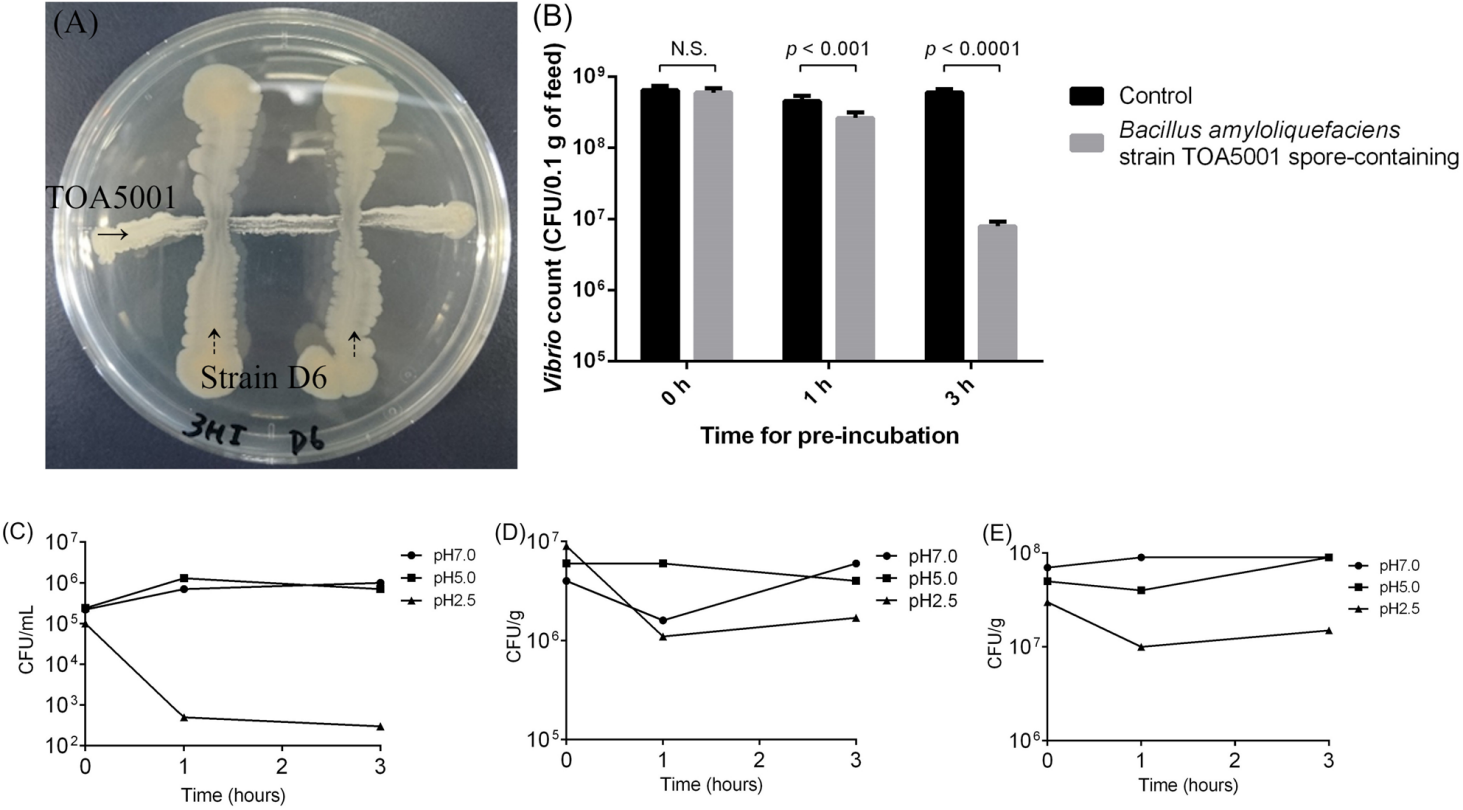
Antagonistic activity and the stability of *Bacillus amyloliquefaciens* strain TOA5001. Inhibition activity of strain TOA5001 against an AHPND-causing strain of *Vibrio parahaemolyticus*, strain D6 was evaluated by cross-streak assay on agar plate (**A**). Growth inhibition of strain TOA5001-containing feed against strain D6 (**B**). Feeds were put in sterile seawater and incubated for 0 h, 1 h and 3 h (pre-incubation), and strain D6 was inoculated. After overnight incubation, the number of bacteria which grew on TCBS agar were counted. Data are presented as mean and vertical bars represent ± SD (n = 6). Statistical analysis was performed using Student’s *t*-test. Stability of strain TOA5001 in different conditions mimicking the shrimp digestive tract for (**C**) the vegetative cells, (**D**) the formulation containing spores and (**E**) the feed containing the formulation of spores.

To test whether *B. amyloliquefaciens* strain TOA5001 has an inhibition activity against an AHPND-causing strain of *V. parahaemolyticus*, we performed in vitro assay. Strain appeared to inhibit the growth of *V. parahaemolyticus* strain D6 on an agar plate (Fig. 6A). We then tested the growth inhibition of *B. amyloliquefaciens* strain TOA5001 against *V. parahaemolyticus* strain D6 in shrimp feed. Shrimp feed with or without spores of strain TOA5001 were put in sterile seawater and incubated for 0 h, 1 h and 3 h (pre-incubation), then strain D6 was inoculated. With at least 1 h of pre-incubation, the growth of D6 was significantly smaller than the controls (Fig. 6B). Also, the stability of strain TOA5001 in different pH conditions was confirmed. Liquid culture (vegetative cells) and spores of strain TOA5001 (formulation) and feed that containing the formulation were subjected to the test of stability in the different pH conditions. Both spores and vegetative cells of strain TOA5001 and strain TOA5001 in the feed were stable at pH 5.0, which was the pH of shrimp stomach, and pH 7.0, while less than 1% survived for 1 h at pH 2.5 (Fig. 6C–E). These results suggested that strain TOA5001 was stable in the stomach of shrimp and the feed containing the spores of strain TOA5001 might moderately inhibit the growth of an AHPND-causing strain of *V. parahaemolyticus* in shrimp.

### Challenge of whiteleg shrimp with *V. parahaemolyticus*

**Figure 7:**
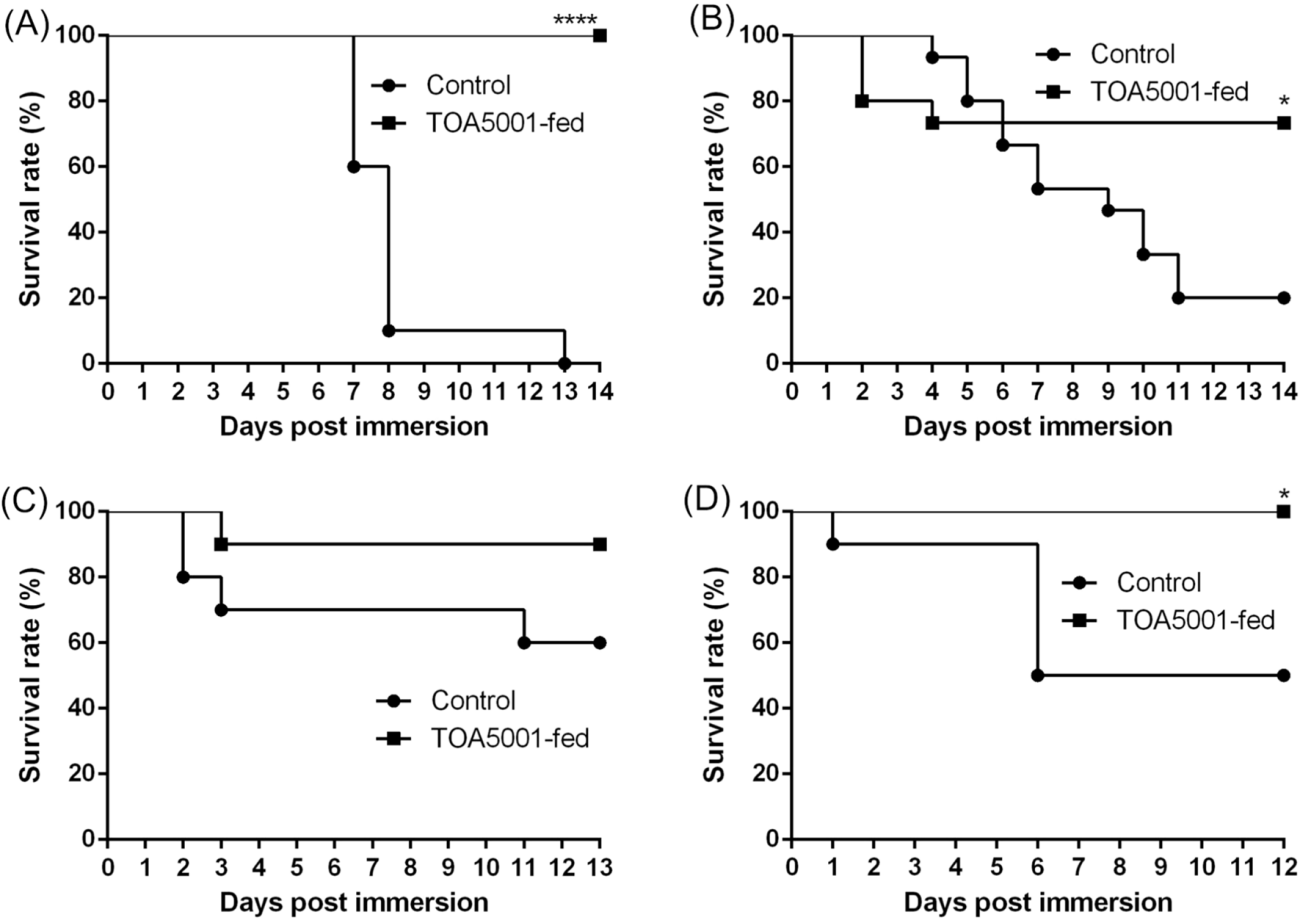
Survival rates of *Litopenaeus vannamei* shrimp after immersion in a tank containing *Vibrio parahaemolyticus* (AHPND-causing strain) at 3 × 10^5^ CFU/mL and after being fed diets with and without probiotic *Bacillus amyloliquefaciens* strain TOA5001. (**A**) and (**B**), first trial. (**A**) After 2 weeks feeding (n = 20). (**B**) After 4 weeks feeding (n = 15) (1st trial). (**C**) and (**D**), second trial. (**C**) After 2 weeks feeding (n = 10). (**D**) After 4 weeks feeding (n = 10). *p < 0.05; ****p < 0.0001 (log‐rank (Mantel–Cox) test, relative to the control group).

We performed challenges on whiteleg shrimp after probiotic-feeding to test the effect of the prevention of AHPND. The shrimp were challenged by adding an AHPND-causing strain of *V. parahaemolyticus* to their tank. In the first trial, the survival rate of the probiotic-fed group was significantly higher (*p* < 0.0001) than that of the control group after 2 weeks of feeding (Fig. 7A), as well as after 4 weeks of feeding (*p* = 0.0205) (Fig. 7B). In the second trial, the survival rate of the probiotic-fed group was slightly higher than that of the control group after 2 weeks of feeding, but the difference was not significant (*p* = 0.1241) (Fig. 7C). However, the survival rate of the probiotic-fed group was significantly higher after 4 weeks of feeding (*p* = 0.0122) (Fig. 7D).

### Microarray analysis

The effect of the probiotic on gene expression of whiteleg shrimp were analyzed by microarray analysis. Of the 15,745 whiteleg shrimp putative genes spotted on the microarray, 88 were differentially expressed between the probiotic-fed and control groups at *p* < 0.05. Of these, 67 genes were upregulated and 21 genes were downregulated in the probiotic-fed shrimp compared to the control (supplementary data, Table S1 and S2). This suggested that the administration might affect the gene expression of whiteleg shrimp, however, it was not clear induction of certain pathways that includes immune responses.

### Effect of probiotic feeding on the numbers of bacteria in the digestive tract

**Figure 8:**
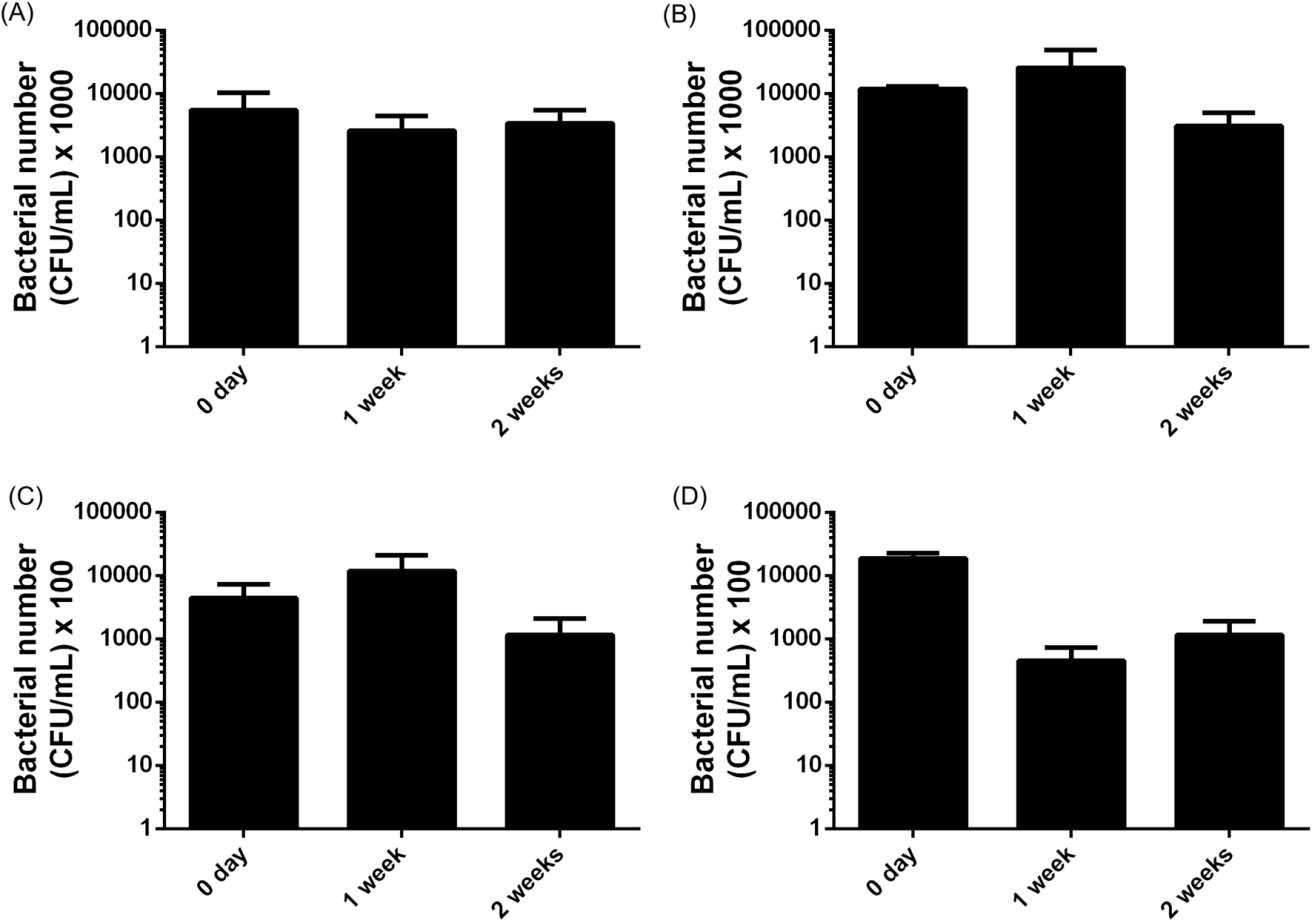
The number of total and *Vibrio* bacteria in the midgut and stomach of whiteleg shrimp fed with feed containing spores of *Bacillus amyloliquefaciens* strain TOA5001 for 1 and 2 weeks. (**A**) Total bacterial number of midgut, (**B**) total bacterial number of stomach, (**C**) number of *Vibrio* bacteria of midgut and (**D**) number of *Vibrio* bacteria of stomach. Data are presented as mean and vertical bars represent ± SD (n = 3). Statistical analysis was performed using Dunn’s multiple comparison test following Kruskal–Wallis test.

To test the actual bacterial number, especially for *Vibrio* species in the digestive tract of whiteleg shrimp. There was no significant difference of the total numbers of bacteria in the midgut and stomach of probiotic-fed shrimp (Fig. 8A,B). There was no statistically significant difference of the number of *Vibrio* bacteria in the midgut and in the stomach of shrimp (for the stomach, *p* = 0.0762 and *p* = 0.2211, compared to the 0 day respectively) (Fig. 8C,D).

## Discussion

A major goal of the current study was to analyze the composition of bacteria which exist in different parts of the digestive tract among two penaeid shrimps, whiteleg shrimp and kuruma shrimp. Interestingly, we found that bacterial diversity was higher in the stomach than in the midgut of both species. This suggests the importance of the analysis of microbiota not only in the midgut, but also in the stomach of shrimp. Also, the relatively higher abundances of *Vibrio* bacteria in the midgut of whiteleg shrimp compared to the stomach, and *Photobacterium* bacteria, potential opportunistic pathogens of shrimp^33^, in the feces of kuruma shrimp compared to the stomach contents suggests that regulation system of bacteria in the stomach. Intestinal microbiota was speculated to be an important factor in shrimp health^2^ based on previous studies of mammalian intestinal microbiota. However, the barrier systems of digestive tract in mammal and invertebrate seem to be different. Intestinal microbiota might be a derived feature of mammal^6^. A peritrophic matrix may result in the protection of midgut rather than microbiota, conversely, specific microbiota in the stomach might be the cause of its higher bacterial diversity. Not knowing the structure of the digestive tract makes it difficult to understand how the shrimp and bacteria interact. Mammal has a mucus layer on the epithelium of gut which harboring indigenous bacteria that colonize on the mucus, working as a barrier against exogenous pathogens^34^. To clarify the colonization of bacteria in the stomach of shrimp, further analyses, including histological studies with specific staining techniques, are needed. Studies of shrimp that live in other conditions, or other species might also elucidate the features of bacteria in the crustacean digestive tract.

Oral feeding of a probiotic changed the bacterial composition in the digestive tract of shrimp. The probiotic diet increased bacterial diversity in the midgut of whiteleg shrimp and in the stomach of kuruma shrimp. Probiotics (a *Bacillus* mix) was reported to affect the diversity of intestinal bacteria of whiteleg shrimp^28^. In the current study, strain TOA5001 of *B. amyloliquefaciens* (a probiotic) seemed to affect the microbiota in both the midgut and stomach, suggesting that it affected the bacterial community throughout the digestive tract. *Isoptericola* bacteria was increased in both midgut and stomach of whiteleg shrimp during probiotic-feeding in this study. However, the interaction of shrimp and *Isoptericola* bacteria and the function have not yet reported so far. On the other hand, Rhodobacteraceae in the stomach of whiteleg shrimp was decreased. Rhodobacteraceae had been reported as potential probiotics, however, the positive and negative effects of Rhodobacteraceae on the growth of shrimp was suggested by Guo et al. (2020)^35^. Because the partial 16S rRNA gene amplicon sequencing cannot accurately distinguish members of Rhodobacteraceae to finer taxonomic levels^36^, the genomic analyses are needed to determine the function of those bacteria on shrimp. Other factors may also affect intestinal bacteria, such as the stage of development and how often the water in the ponds is changed^37^. However, whiteleg shrimp raised under laboratory conditions and kuruma shrimp raised in an aquaculture farm, i.e. different species and different conditions, were affected similarly by the probiotic. The effects of probiotics on bacterial diversity in the digestive tract and shrimp health remain unclear, although healthy shrimp were found to have a higher bacterial diversity in the intestine^38^ and diseased shrimp were found to have lower bacterial diversity in the stomach^23^ and intestine^39^.

Another goal of this study was to prevent shrimp death caused by an AHPND strain of *V. parahaemolyticus* using a probiotic. Shrimp that were fed *B. amyloliquefaciens* strain TOA5001 showed significantly higher survival compared to the control after the challenge with an AHPND strain of *V. parahaemolyticus*, strain D6. The digestive tract of shrimp is one of the main routes of pathogen infection. According to previous studies, pathogenic bacteria including AHPND strains of *V. parahaemolyticus* colonize in shrimp stomach and cause shrimp death^21,40^, suggesting the importance of microbiota in the stomach^23^. The use of probiotics is thought to be a promising approach for sustainable aquaculture by preventing diseases and reducing the use of antibiotics^25,26,41^. There are some possible mechanisms by which probiotics inhibit pathogenic bacteria in shrimp include competing with pathogenic bacteria, producing antimicrobial substances^42^, having immunostimulatory effects^43^ and manipulating the intestinal microbiota^28^. However, to date, the effects of probiotics on the stomach microbiota of shrimp and disease prevention are unclear. Shrimp fed *Bacillus* spores were found to have higher mRNA levels of immune-related genes, and a higher survival rate after being challenged with a virulent bacterium^44^. In addition, immune-related genes were found to be upregulated in stomach of shrimp during *V. parahaemolyticus* infection^45^. This suggests immune stimulation that caused by probiotics might be a potential approach to prevent AHPND. However, in the current study, strain TOA5001 did not affect the mRNA levels of immune-related genes, suggesting that strain TOA5001 prevented AHPND by some means other than immune stimulation. Extracts from *B. subtilis*-fermented soybean were reported to inhibit *Vibrio* biofilm formation and to reduce the mortality of shrimp challenged by a pathogenic strain of *Vibrio harveyi*, but they had no bactericidal effect^46^. Similarly, the substance(s) produced by strain TOA5001 did not kill strain D6, but it did moderately inhibit its growth. Antagonistic activity of strain TOA5001 may inhibit invasion of AHPND-causing *V. parahaemolyticus* in shrimp. Further studies on the interaction and the mechanism of exogenous probiotics and pathogenic *Vibrio* bacteria in the digestive tract of shrimp are needed. Appropriate control groups for experiments including feeding of inactivated spores may contribute to this.

In mammalian models, it was difficult to colonize the gut with probiotics^47^, even though dietary *Bacillus* spores were found to inhibit pathogens^48^. In shrimp, colonization of exogenous probiotics in the digestive system has not been analyzed. Because invertebrates lack gastric acid, their digestive systems have slightly acidic to neutral pH^3^. The pH in the digestive gland of penaeid shrimps, including whiteleg shrimp was found to be 5.7 ± 0.1^49^. In this study, both the spores and vegetative cells of strain TOA5001 were stable at that pH. Strain TOA5001 was also isolated from fecal matter of shrimp being fed strain TOA5001. However, after the feeding of strain TOA5001 was stopped, the bacteria disappeared from shrimp intestine (data not shown). Moreover, in the analysis of 16S rRNA genes of the digestive tract of shrimp, it was difficult to find the reads of strain or the species from the data. These results indicated that strain TOA5001 stayed in the digestive tract of shrimp only for a short period. Although further studies are needed to clarify the kinetics of exogenous probiotics in the digestive tract, continuous administration of probiotic strains may be needed to inhibit pathogenic bacteria of shrimp.

In summary, bacterial diversity was found to be higher in the stomach than in the midgut in both penaeid shrimps, whether or not they were fed probiotics. Our results also suggest that probiotics increased bacterial diversity, distributed microbiota throughout the digestive tract, and, in whiteleg shrimp, prevented AHPND. The difference in the digestive systems of mammals and invertebrates should result in differences in host–bacteria interactions. Further studies of shrimp–bacteria interactions in the gut will contribute to a better understanding of barrier immunity in the digestive tract as well as a more sustainable aquaculture.

## Methods

### Shrimps and bacterial strains

Whiteleg shrimp were maintained in 100-L plastic tanks containing artificial seawater. Salinity was adjusted to 30 ppt and the temperature was kept in the range 27–29 °C during the following experiments. A Totto filter system (Bio Labo Totto Co., Ltd., Japan) was placed on the tank. Evaporated water was replaced with fresh water. Fecal matter on the bottoms of the tanks was removed when required. Before the experiments, shrimp were fed commercial feed on a daily basis.

Kuruma shrimp were cultured in concrete ponds in a kuruma shrimp farm in Kume-island, Okinawa, Japan.

*Bacillus amyloliquefaciens* strain TOA5001^50^ and spores of strain TOA5001 at a concentration of 1 × 10^8^ colony-forming units (CFU)/g in rice bran^50^ (the formulation) was kindly provided by TOA BIOPHARMA CO., LTD. (Tokyo, Japan) and used in all the experiments.

*Vibrio parahaemolyticus* strain D6^51^, an AHPND-causing strain isolated in Thailand, was used in the challenge test and inhibition assay. Culture broth of strain D6 was mixed with glycerol and stored at − 80 °C for challenge tests.

### Feed preparation

Feed for whiteleg shrimp was prepared follows. Commercial whiteleg shrimp feed was powdered using a food-processor. A formulation containing *B. amyloliquefaciens* strain TOA5001 at 1 × 10^8^ CFU/g was mixed thoroughly with the powdered feed at 5% (w/w). The same weight of water was mixed with the feed to form a mash, which was then squeezed into a ball. Then, the feed was pressed out to make narrow cylindrical form and dried completely at 60 °C. The final feed contained 5 × 10^6^ CFU/g of *B. amyloliquefaciens* strain TOA5001. The control feed was prepared in the same way except without strain TOA5001. The experimental feeds were stored at 4 °C until use.

The effect of pH on the viability of strain TOA5001 was analyzed. To measure stomach pH, shrimp fed commercial feed were dissected 30 min after the start of feeding. pH test paper placed on the stomach epithelium indicated the pH was 5.0. Heart infusion (HI) liquid media containing 3% NaCl were adjusted to pH 5.0 and pH 2.5 using HCl. Strain TOA5001 was cultured in the HI media until the densities reached 1 × 10^7^ CFU/mL. To obtain vegetative cells, the broths were centrifuged, and the supernatants were removed. Aliquots (0.01 g) of the strain TOA5001 formulation and the feed containing strain TOA5001 were put in centrifuge tubes. One millilitre of the pH-adjusted media (pH 5.0 or pH 2.5) was added to the centrifuge tubes. The centrifuge tubes were incubated at 28 °C with shaking. The number of live cells was expressed as the number of colonies that grew on HI agar plates as described previously^52^.

The experiments on kuruma shrimp were conducted in a commercial shrimp farm. The feed for the kuruma shrimp used in the experiments was prepared by a feed company using the same formulation used for whiteleg shrimp. Its final concentration of *B. amyloliquefaciens* strain TOA5001 was 2.4 × 10^5^ CFU/g.

### Analysis of microbiota of the digestive tract of whiteleg shrimp

Whiteleg shrimp (approximately 2.0 g each) were fed daily at 5% of body weight for two and four weeks, fasted for 24 h, dipped in 75% ethanol and washed with sterile artificial seawater twice to minimize contamination of environmental microorganisms on the body surface. The stomach and midgut were put in centrifuge tubes and stored at − 80 °C.

Total DNA was extracted from each tissue using lysis enzymes and the CTAB-method. Briefly, 180 µL of lysozyme solution containing 20 mg/mL of chicken lysozyme, 20 mM Tris–HCl (pH 8.0) and 1% SDS was added and tissue was homogenized thoroughly. After incubation at 37 °C for 1 h, 500 µL of lysis solution (0.1 M EDTA (pH 8.0) and 1% SDS) and 5 µL of proteinase K (20 mg/mL) were added and incubated at 55 °C overnight. CTAB solution (10% CTAB and 0.7 M NaCl) and 165 µL of 5 M NaCl was added and incubated at 55 °C for 10 min. One millilitre of PCI (phenol:chloroform:isoamyl alcohol = 25:24:1) (pH 8.0) was added and mixed. Centrifugation was conducted and the supernatant was collected. DNA in the supernatant was precipitated using isopropanol and washed in ethanol, then dissolved in TE buffer [10 mM Tris–HCl and 1 mM EDTA (pH 8.0)]. RNA was eliminated by digestion with RNase treatment overnight, then, DNA was precipitated using ethanol with sodium acetate and dissolved in TE buffer again. The concentration of DNA was measured using spectrophotometer, NanoDrop Lite (Thermo Scientific, USA) and adjusted at 1.0 ng/µL.

Purified total DNA was used to amplify the partial sequence of 16S rRNA gene in 30 µL of the reaction mix (1.5 µL of template DNA, 3.0 µL of 10 × Ex Taq Buffer, 2.4 µL of dNTP Mix (2.5 mM each), 0.6 µL of forward and reverse primer (10 µM), 0.15 µL of TaKaRa Ex Taq (Takara Bio, Japan), 21.75 µL of sterilized Milli-Q water). The primer pair 515F (5′-**TCGTCGGCAGCGTCAGATGTGTATAAGAGACAG**GTGCCAGCMGCCGCGGTAA-3′) and 806R (5′-**GTCTCGTGGGCTCGGAGATGTGTATAAGAGACAG**GGACTACHVGGGTWTCTAAT-3′) corresponding to the V4 region of the 16S rRNA gene with Illumina Nextera adapters (in bold) was used. The reaction was 30–35 cycles depending on the amplification. The PCR amplicons were confirmed using agarose gel-electrophoresis. Further preparation of samples for sequencing with Illumina Miseq, including index application and purification and library construction by using Nextera XT DNA Library Preparation Kit were conducted following the protocol that provided by the company Illumina, ‘16S Metagenomic Sequencing Library Preparation’^53^. The PCR amplicons with index sequences were confirmed and the molarity was measured using D1000 ScreenTape System (Agilent Technologies, USA) and the concentration of DNA was measured using Qubit dsDNA HS Assay Kit (Invitrogen, USA). The purified libraries were subjected to the 2 × 150 bp sequencing run on Miseq (Illumina, USA).

Sequence data in FASTQ format were analyzed using Quantitative Insights into Microbial Ecology 2 (QIIME 2)^54^ (https://qiime2.org/). Reads were denoised, filtered with DADA2 plugin. The sequences were classified using SILVA 132 database based on 99% of identity. The taxonomy with 6 levels (i.e. Genus levels) was used to generate bar plots of relative abundance of different taxa in Qiime2 View^55^ (https://view.qiime2.org/). Principal coordinate analysis (PCoA) was performed using Qiime2 and visualized using Emperor^56^. Alfa diversity was analyzed using the number of amplicon sequence variants (ASVs) and Shannon index, calculated in Qiime2 with sequence depth at 30,000 for each sample. Boxplot figures for alfa diversity were then prepared using GraphPad Prism 6 (GraphPad, USA) and statistically analyzed using Mann–Whitney test, or Dunn’s multiple comparison test following Kruskal–Wallis test in GraphPad Prism. Note that, the paired-end reads were analyzed as single-end reads because those reads were obtained using 2 × 150 bp running on Miseq, causing an error on DADA2 during the merging step. DADA2 requires 20 bp or more overlaps between the paired-end sequences.

### Analysis of microbiota of the digestive tract of kuruma shrimp

Kuruma shrimp (approximately 25 g each) were harvested from the ponds, placed in cool seawater to calm them and transported to the laboratory in Tokyo. Transportation took about 24 h. Feces and stomach contents were collected, placed in centrifuge tubes and stored at − 80 °C.

DNA were extracted from feces and stomach contents using QIAamp DNA stool mini kit (QIAGEN, Germany) following the manufactured protocol. DNA yield was measured using a NanoDrop lite and adjusted at 1.0 ng/µL.

Purified total DNA was used to amplify the partial sequence of 16S rRNA gene using the same methods used for whiteleg shrimp above.

Raw sequence reads were analyzed using QIIME2 and graphs were made using QIIME2 View with the same methods used above.

### Challenge test

Oral infection is thought to be the main route of invasion of AHPND strains of *V. parahaemolyticus*^14,21,22^. Further, immersion is recommended for bioassay challenge of AHPND^57^. Therefore, we conducted the challenge test by immersion infection rather than injection. Whiteleg shrimp were fed the experimental and control diets at a rate of 5% body weight per day, divided into three equal feedings each day. After feeding for 2 and 4 weeks, shrimp were transferred to aerated aquarium tanks containing 10 L of artificial seawater, without a filter system. AHPND strain D6 of *V. parahaemolyticus* was added to the water at a final concentration of 3 × 10^5^ CFU/mL. The mortality of shrimp was then observed. The shrimp were fed the experimental diets throughout the challenge test. Strain D6 was judged as the cause of death of all shrimp that died. The experiment was conducted twice. Figures were produced, and the data were analyzed using GraphPad Prism. Survival was analyzed using the Kaplan–Meier method and significant difference was evaluated by log‐rank (Mantel–Cox) test, relative to the control group.

### Activity of strain TOA5001 against *V. parahaemolyticus* strain D6

Strain TOA5001 was inoculated on HI agar plates and cultured at 30 °C. Strain D6 was inoculated on a HI agar plate containing 3% of NaCl and incubated at 30 °C. Using the cross-streak method^58^, strain D6 was streaked on an HI agar plate containing 3% of NaCl, and then the plates were cross-steaked with strain TOA5001 and incubated at 30 °C.

The feed containing strain TOA5001 and the control feeds were put in 500 µL of sterile artificial seawater and incubated at 30 °C for 0 h, 1 h and 3 h (pre-incubation for germination of spores). Strain D6 was inoculated to the feeds and incubated overnight. The number of bacterial colonies that grew on TCBS agar was counted.

### Microarray analysis

Microarray analysis was conducted by the method of Pedrosa-Gerasmio et al.^59^. Total RNA was extracted from the hepatopancreas of shrimp (n = 4) after 2 weeks of feeding, using RNAiso Plus (Takara Bio, Japan) following the manufacturer’s protocol. Residual DNA was eliminated by digestion with DNase. RNA quality was measured with a Qubit 2.0 fluorometer (Invitrogen, USA). Samples were prepared following the One-Color Microarray-Based Gene Expression Analysis protocol (Agilent Technologies, USA). Total RNA (200 ng) was reverse transcribed with RNA spike-in controls and labeled with Cyanine-3 CTP using a Low Input Quick Amp Labelling Kit, One Color (Agilent Technologies, USA). Hybridization was done for 17 h at 65 °C and a Custom Gene Expression Microarray GE 8 × 15 k (Agilent Technologies, USA.) containing 15,745 probes for known genes and expressed sequence tags from *L. vannamei*^40^. The hybridized slide was washed and scanned immediately using a DNA Microarray Scanner with SureScan High-Resolution Technology (Agilent Technologies, USA). Data were extracted from the scanned slides using Agilent Feature Extraction Software 10.7.3.1 using the default parameters. To determine differentially expressed genes (DEGs), data were log- transformed, normalized, and analyzed using Subio Platform software (Subio Inc., Japan). DEGs with fourfold difference between the probiotic-fed and the control were used for t test analysis with *p* < 0.05. BLASTX searches were performed in Blast2GO (http://www.blast2go.org/).

### Numbers of bacteria in the digestive tract

Whiteleg shrimp were fed probiotic-containing feed for up to 2 weeks. Before starting the administration of the probiotic, after 1 and 2 weeks of feeding, midgut and stomach were collected and put in 500 µL of sterilized artificial seawater. The samples were homogenized. HI agar plates containing either 3% NaCl or thiosulfate citrate bile salts sucrose (TCBS) were inoculated with drops of serially diluted homogenate to count total bacteria and *Vibrio* bacteria, respectively. After overnight incubation, bacterial colonies were counted. Figures were produced, and the data were analyzed using Dunn’s multiple comparison test following Kruskal–Wallis test in GraphPad Prism.

## Supplementary Information

Supplementary Tables.
