# Supplementary material for: Analysis of microbiota in the stomach and midgut of two penaeid shrimps during probiotic feeding
Source: Sci Rep. 2021 May 11;11:9936. doi: 10.1038/s41598-021-89415-w (PMC8113331; doi:10.1038/s41598-021-89415-w)
Supplement: Supplementary file 1 — Supplementary Tables. [file 41598_2021_89415_MOESM1_ESM.pdf]

Supplementary Data

Analysis of microbiota in the stomach and midgut of two penaeid shrimps during probiotic feeding

Kentaro Imaizumi[a], Sasiwipa Tinwongger[a, b], Hidehiro Kondo[a] and Ikuo Hirono[a*]

[a]*Laboratory of Genome Science, Tokyo University of Marine Science and Technology, Tokyo, Japan*

[b]*Department of Fisheries, Kasetklang Chatuchak, Bangkok, 10900, Thailand*

Corresponding author

Ikuo Hirono, Laboratory of Genome Science, Graduate School of Marine Science and Technology, Tokyo University of Marine Science and Technology, 4-5-7 Konan, Minato, Tokyo, 108-8477, Japan (e-mail: hirono@kaiyodai.ac.jp)

Table S1. Sequence description of differentially expressed genes with more than fourfold difference (*t* test, $p < 0.05$) between *Bacillus amyloliquefaciens* strain TOA5001-fed and the control groups with their fold change values; higher mRNA levels in *Bacillus amyloliquefaciens* strain TOA5001-fed shrimps (n = 4).

Prob name	Blast top hit	Accession # of top hit	Fold change
gnl|UG|Lva_S62262313	hypothetical protein C7M84_011899 [Penaeus vannamei]	ROT85553.1	7.4541836
gnl|UG|Lva_S62262294	Kinesin-related protein 1, partial [Penaeus vannamei]	ROT74429.1	4.66564
gnl|UG|Lva_S46129573	chorion peroxidase-like [Penaeus vannamei]	XP_027228241.1	7.83931
gnl|UG|Lva_S62270261	hypothetical protein C7M84_011513 [Penaeus vannamei]	ROT70231.1	4.2284617
gnl|UG|Lva_S62240698	pregnancy zone protein-like [Penaeus vannamei]	XP_027226646.1	6.2210174
gnl|UG|Lva_S62242914	phospholipase C, beta isoform [Penaeus vannamei]	ROT67518.1	5.0604315
gnl|UG|Lva_S62254179	limbic system-associated membrane protein-like [Penaeus vannamei]	XP_027229379.1	5.4594765
gnl|UG|Lva_S62236725	protein singed-like isoform X3 [Penaeus vannamei]	XP_027210079.1	5.914224
gnl|UG|Lva_S62245645	PREDICTED: GTP cyclohydrolase 1-like [Hyalella azteca]	XP_018008294.1	17.161297
gnl|UG|Lva_S62303778	uncharacterized protein LOC113803732 [Penaeus vannamei]	XP_027210344.1	4.2569766
gnl|UG|Lva_S62255842	RING-type E3 ubiquitin-protein ligase PPIL2-like [Penaeus vannamei]	XP_027229175.1	4.277465
gnl|UG|Lva_S46161202	protein ALP1-like [Penaeus vannamei]	XP_027228201.1	4.159256
gnl|UG|Lva_S62233160	NF-kappa-B inhibitor cactus-like [Penaeus vannamei]	XP_027232117.1	5.013407
gnl|UG|Lva_S46211088	NADH dehydrogenase [ubiquinone] 1 alpha subcomplex subunit 5-like [Penaeus vannamei]	XP_027228433.1	5.6134353
gnl|UG|Lva_S62265035	guanine nucleotide exchange factor for Rab-3A-like [Penaeus vannamei]	XP_027216738.1	4.0216045
gnl|UG|Lva_S62266699	ADAM 17-like protease [Penaeus vannamei]	XP_027207790.1	4.319845
gnl|UG|Lva_S62270076	WD repeat-containing protein 20-like [Penaeus vannamei]	XP_027213005.1	4.5225954
gnl|UG|Lva_S62263281	oocyte zinc finger protein XlCOF6.1-like isoform X1 [Nilaparvata lugens]	XP_022190664.1	7.841732
gnl|UG|Lva_S62241511	transient receptor potential channel pyrexia-like [Penaeus vannamei]	XP_027237392.1	4.5393915
gnl|UG|Lva_S62255753	potassium voltage-gated channel protein Shaker-like [Penaeus vannamei]	XP_027234367.1	8.286181
gnl|UG|Lva_S62259387	ryanodine receptor [Penaeus vannamei]	ROT85518.1	9.395655
gnl|UG|Lva_S46130595	small integral membrane protein 4-like [Penaeus vannamei]	XP_027213130.1	4.2011523
gnl|UG|Lva_S62247511	adenosine deaminase-like [Penaeus vannamei]	XP_027238533.1	4.3525577
gnl|UG|Lva_S62235451	LOW QUALITY PROTEIN: kinase D-interacting substrate of 220 kDa B-like [Penaeus vannamei]	XP_027227604.1	10.274463

gnl|UG|Lva_S62262245	putative sideroflexin 1,2,3 [Penaeus vannamei]	ROT80286.1	4.229677
gnl|UG|Lva_S62243451	Vesicular acetylcholine transporter [Armadillidium vulgare]	RXG60485.1	6.203374
gnl|UG|Lva_S62273420	extracellular matrix protein FRAS1-like [Penaeus vannamei]	XP_027218020.1	4.8090734
gnl|UG|Lva_S62255461	uncharacterized protein LOC113808297 isoform X1 [Penaeus vannamei]	XP_027215466.1	6.278118
gnl|UG|Lva_S62256684	uncharacterized protein LOC113827724, partial [Penaeus vannamei]	XP_027236418.1	4.4257593
gnl|UG|Lva_S62300068	LOW QUALITY PROTEIN: Ig-like and fibronectin type-III domain-containing protein 2 [Penaeus vannamei]	XP_027230115.1	4.0408998
gnl|UG|Lva_S62297574	hypothetical protein C7M84_010411 [Penaeus vannamei]	ROT71272.1	4.3757133
gnl|UG|Lva_S62278146	gamma-butyrobetaine dioxygenase-like isoform X2 [Penaeus vannamei]	XP_027236776.1	5.0600615
gnl|UG|Lva_S62249246	suppressor of cytokine signaling-2 like protein [Penaeus vannamei]	ROT60867.1	4.067962
gnl|UG|Lva_S62279239	hypothetical protein C7M84_002655 [Penaeus vannamei]	ROT78643.1	4.095677
gnl|UG|Lva_S62305727	LOW QUALITY PROTEIN: transmembrane and TPR repeat-containing protein CG4050-like [Penaeus vannamei]	XP_027209697.1	14.029534
gnl|UG|Lva_S58726390	fatty acid synthase [Penaeus vannamei]	ADM88556.1	4.3802505
gnl|UG|Lva_S62303147	PREDICTED: heparan sulfate 2-O-sulfotransferase pipe-like [Hyalella azteca]	XP_018011496.1	5.5796957
gnl|UG|Lva_S62241355	F-box/LRR-repeat protein 7-like [Penaeus vannamei]	XP_027212195.1	4.560883
gnl|UG|Lva_S62303507	peroxisomal leader peptide-processing protease-like [Penaeus vannamei]	XP_027227876.1	5.2403345
gnl|UG|Lva_S62305627	LOW QUALITY PROTEIN: delta and Notch-like epidermal growth factor-related receptor [Penaeus vannamei]	XP_027237037.1	5.266432
gnl|UG|Lva_S62268537	PRA1 family protein 3-like [Penaeus vannamei]	XP_027228379.1	15.675887
gnl|UG|Lva_S62245719	E3 ubiquitin-protein ligase hyd [Penaeus vannamei]	ROT64339.1	5.4174294
gnl|UG|Lva_S62263473	muscle calcium channel subunit alpha-1-like [Penaeus vannamei]	XP_027218824.1	5.389818
gnl|UG|Lva_S62298240	LOW QUALITY PROTEIN: uncharacterized protein LOC113804390 [Penaeus vannamei]	XP_027211056.1	6.5257397
gnl|UG|Lva_S62275614	hypothetical protein C7M84_024534 [Penaeus vannamei]	ROT82296.1	12.8725815
gnl|UG|Lva_S62247353	ATP-dependent (S)-NAD(P)H-hydrate dehydratase-like [Penaeus vannamei]	XP_027214949.1	5.511696
gnl|UG|Lva_S62267995	LOW QUALITY PROTEIN: sodium channel protein para-like [Penaeus vannamei]	XP_027236630.1	6.0556703
gnl|UG|Lva_S62246351	DNA polymerase alpha catalytic subunit-like [Penaeus vannamei]	XP_027207566.1	4.45532
gnl|UG|Lva_S62265150	protein diaphanous-like isoform X2 [Penaeus vannamei]	XP_027213745.1	4.4288793
gnl|UG|Lva_S62245162	gamma-glutamyl hydrolase-like [Penaeus vannamei]	XP_027238320.1	4.490572
gnl|UG|Lva_S62306455	PREDICTED: tetratricopeptide repeat protein 30A-like [Hyalella azteca]	XP_018016574.1	4.077428
gnl|UG|Lva_S62299763	ankyrin repeat and SAM domain-containing protein 4B-like [Folsomia candida]	XP_021951083.1	4.4300213
gnl|UG|Lva_S62282426	myosin heavy chain type 6a [Penaeus vannamei]	ROT74108.1	8.489346
gnl|UG|Lva_S62265995	myosin heavy chain type 2 [Penaeus vannamei]	ROT72880.1	18.37178
gnl|UG|Lva_S62304306	hypothetical protein C7M84_017924 [Penaeus vannamei]	ROT64151.1	5.899616

gnl|UG|Lva_S62262523	calcium uptake protein 1 homolog, mitochondrial-like isoform X1 [Penaeus vannamei]	XP_027210033.1	5.6974688
gnl|UG|Lva_S62256925	LOW QUALITY PROTEIN: transferrin-like [Penaeus vannamei]	XP_027215079.1	4.0959706
gnl|UG|Lva_S62267891	hypothetical protein C7M84_002289, partial [Penaeus vannamei]	ROT78981.1	4.9314423
gnl|UG|Lva_S62255960	Sarcolemmal membrane-associated protein [Penaeus vannamei]	ROT78483.1	7.045258
gnl|UG|Lva_S62255641	sec1 family domain-containing protein 1-like [Penaeus vannamei]	XP_027229589.1	4.242684
gnl|UG|Lva_S46258482	LOW QUALITY PROTEIN: ovoinhibitor-like [Penaeus vannamei]	XP_027210484.1	23.252996
gnl|UG|Lva_S62279017	protein krueppel-like [Penaeus vannamei]	XP_027222685.1	8.202782
gnl|UG|Lva_S62303496	LOW QUALITY PROTEIN: rho-related BTB domain-containing protein 1-like [Penaeus vannamei]	XP_027230766.1	4.352234
gnl|UG|Lva_S62256704	suppressor of fused homolog isoform X3 [Penaeus vannamei]	XP_027217122.1	4.4131207
gnl|UG|Lva_S47502078	type II hemocyte transglutaminase [Penaeus vannamei]	ABX83902.1	4.849655
gnl|UG|Lva_S62269138	Ecdysone-induced protein 74EF isoform B [Penaeus vannamei]	ROT82945.1	4.5178704
gnl|UG|Lva_S62258776	exosome RNA helicase MTR4-like isoform X1 [Penaeus vannamei]	XP_027239414.1	4.038301

Table S2. Sequence description of differentially expressed genes with more than fourfold difference (t test, $p < 0.05$) between *Bacillus amyloliquefaciens* strain TOA5001-fed and the control groups with their fold change values; lower mRNA levels in *Bacillus amyloliquefaciens* strain TOA5001-fed shrimps (n = 4).

Prob name	Blast top hit	Accession	Fold change
gnl|UG|Lva_S46162788	40S ribosomal protein S28 [Armadillidium vulgare]	RXG56747.1	0.24694455
gnl|UG|Lva_S46190674	GTP-binding protein SAR1-like isoform X1 [Penaeus vannamei]	XP_027232436.1	0.104448974
gnl|UG|Lva_S62245558	dynamin-like 120 kDa protein, mitochondrial [Penaeus vannamei]	XP_027238384.1	0.20341234
gnl|UG|Lva_S46122538	dimethyladenosine transferase 1, mitochondrial-like isoform X1 [Penaeus vannamei]	XP_027213459.1	0.23895314
gnl|UG|Lva_S46135988	DD9A, partial [Penaeus japonicus]	BAA90875.1	0.18496639
gnl|UG|Lva_S62242586	rho GTPase-activating protein 26-like isoform X11 [Penaeus vannamei]	XP_027217614.1	0.24103487
gnl|UG|Lva_S62296874	liver carboxylesterase 2-like isoform X1 [Penaeus vannamei]	XP_027238198.1	0.22687602
gnl|UG|Lva_S62243124	F-box-like/WD repeat-containing protein TBL1X [Diachasma alloeum]	XP_015121402.1	0.24156831
gnl|UG|Lva_S62261565	uncharacterized protein LOC113804202 [Penaeus vannamei]	XP_027210834.1	0.22957987
gnl|UG|Lva_S62247082	carboxylase:pyruvate/acetyl-coa/propionyl-CoA [Penaeus vannamei]	ROT68219.1	0.17822921
gnl|UG|Lva_S62241750	Thyroid receptor-interacting protein 11, partial [Armadillidium vulgare]	RXG70218.1	0.23409212
gnl|UG|Lva_S62246299	ubiquinone biosynthesis O-methyltransferase, mitochondrial-like [Penaeus vannamei]	XP_027211585.1	0.10147309
gnl|UG|Lva_S62256827	hypothetical protein C7M84_018546 [Penaeus vannamei]	ROT63572.1	0.1041738
gnl|UG|Lva_S62297549	hypothetical protein C7M84_014008 [Penaeus vannamei]	ROT67891.1	0.20164768
gnl|UG|Lva_S46154756	F-box only protein 44-like isoform X1 [Penaeus vannamei]	XP_027219715.1	0.098679036
gnl|UG|Lva_S62245587	torso-like protein isoform X2 [Penaeus vannamei]	XP_027220879.1	0.21490553
gnl|UG|Lva_S62275760	tyrosine-protein phosphatase non-receptor type 9-like isoform X2 [Penaeus vannamei]	XP_027235701.1	0.22801955
gnl|UG|Lva_S46199864	polysaccharide deacetylase family protein [Gammaproteobacteria bacterium 2W06]	WP_110883485.1	0.20890088
gnl|UG|Lva_S62267724	ras-related GTP-binding protein C-like [Penaeus vannamei]	XP_027213531.1	0.15758643
gnl|UG|Lva_S46154104	LOW QUALITY PROTEIN: maspardin-like [Penaeus vannamei]	XP_027217188.1	0.23935133
gnl|UG|Lva_S46253092	aspartate beta-hydroxylase domain-containing protein 2-like [Penaeus vannamei]	XP_027239286.1	0.12172425
